# The Genomes of Two Strains of *Taenia crassiceps* the Animal Model for the Study of Human Cysticercosis

**DOI:** 10.3389/fcimb.2022.876839

**Published:** 2022-05-10

**Authors:** Raúl J. Bobes, Karel Estrada, Diana G. Rios-Valencia, Arturo Calderón-Gallegos, Patricia de la Torre, Julio C. Carrero, Alejandro Sanchez-Flores, Juan P. Laclette

**Affiliations:** ^1^ Biomedical Research Institute, Universidad Nacional Autónoma de México, CDMX, Mexico; ^2^ Biotechnology Institute, Universidad Nacional Autónoma de México, Cuernavaca, Mexico

**Keywords:** *Taenia crassiceps*, ORF, WFU, comparative genomics, RNA-seq, differential expression, cysticercosis, animal model

## Abstract

Human cysticercosis by *Taenia solium* is the major cause of neurological illness in countries of Africa, Southeast Asia, and the Americas. Publication of four cestode genomes (*T. solium*, *Echinococcus multilocularis*, *E. granulosus* and *Hymenolepis microstoma*) in the last decade, marked the advent of novel approaches on the study of the host-parasite molecular crosstalk for cestode parasites of importance for human and animal health. *Taenia crassiceps* is another cestode parasite, closely related to *T. solium*, which has been used in numerous studies as an animal model for human cysticercosis. Therefore, characterization of the *T. crassiceps* genome will also contribute to the understanding of the human infection. Here, we report the genome of *T. crassiceps* WFU strain, reconstructed to a noncontiguous finished resolution and performed a genomic and differential expression comparison analysis against ORF strain. Both strain genomes were sequenced using Oxford Nanopore (MinION) and Illumina technologies, achieving high quality assemblies of about 107 Mb for both strains. Dotplot comparison between WFU and ORF demonstrated that both genomes were extremely similar. Additionally, karyotyping results for both strains failed to demonstrate a difference in chromosome composition. Therefore, our results strongly support the concept that the absence of scolex in the ORF strain of *T. crassiceps* was not the result of a chromosomal loss as proposed elsewhere. Instead, it appears to be the result of subtle and extensive differences in the regulation of gene expression. Analysis of variants between the two strains identified 2,487 sites with changes distributed in 31 of 65 scaffolds. The differential expression analysis revealed that genes related to development and morphogenesis in the ORF strain might be involved in the lack of scolex formation.

## Introduction

The tapeworm family Taeniidae has been extensively studied, particularly because of the importance of *Taenia solium*, *Taenia asiatica* (Wang et al., 2016; Gripper and Welburn, 2017), *Echinococcus granulosus* and *Echinococcus multilocularis* (Battelli, 2009; Deplazes et al., 2017) as public health threats. Other species also have veterinary importance such as *Taenia saginata* and *Taenia pisiformis* (Silva and Costa-Cruz, 2012; Yang et al., 2012).

*T. solium* is the cestode parasite that generates a more significant economic and health burden on humans and pigs, this parasite, is responsible of porcine cysticercosis, human teniasis and neurocysticercosis, considered the most frequent parasite disease of the central nervous system (Garcia and Modi, 2008; Fleury et al., 2010; Carpio et al., 2018), and a neglected tropical disease by the World Health Organization (World Health Organization, 2011). Humans acquire cysticercosis through fecal-oral contamination with *T. solium* eggs from human tapeworm carriers. The most common places for the establishment of the larval form in the intermediate host are the skeletal muscles, the ocular system and the central nervous system. Cysticercosis is of great economic and public health importance, causing morbidity and mortality in countries and regions with deficient sanitary conditions. Many of these developing countries are located in Southeast Asia, Africa and the Americas (Ito et al., 2003; Flisser et al., 2006; Lustigman et al., 2012; Zammarchi et al., 2013; Ito, 2015). In the last decades, cysticercosis has spread to Europe, the United States and Australia, mainly due to the increase in human migration from endemic regions (Gabriël et al., 2015; O’Neal and Flecker, 2015). *T. solium* has been globally classified as the main foodborne parasite by the United Nations Food and Agriculture Organization (FAO) in 2014 and the World Health Organization (Torgerson et al., 2015).

*Taenia crassiceps*, a close relative of *T. solium*, can be easily maintained in most animal facilities and the cysticerci can be manipulated *in vivo* and *in vitro*, providing a suitable model for the study of several aspects of human cysticercosis (Willms and Zurabian, 2010). Thus, the intraperitoneal murine infection by *T. crassiceps* has been widely used as a model of cysticercosis in studies of the host immune response, diagnosis, evaluation of anthelmintic drugs, vaccine development (Larralde et al., 1990; Toledo et al., 2001; Vargas-Villavicencio et al., 2005; Sciutto et al., 2011; Mahanty et al., 2013); sex and genetic influence on the susceptibility to infection (Sciutto et al., 1991), influence of the parasite on the hormonal status of the infected host (Morales-Montor et al., 2008), development of practical methods for transfection of exogenous genes (Moguel et al., 2015), among other aspects. Besides, *T. crassiceps* infections in humans are very rare, isolated cases have been reported in immunosuppressed individuals (Heldwein et al., 2006; Willms and Zurabian, 2010; Lescano and Zunt, 2013; Deplazes et al., 2019). Human infections by *T. crassiceps* include subcutaneous and intramuscular (François et al., 1998; Maillard et al., 1998; Heldwein et al., 2006; Goesseringer et al., 2011), intraocular (Shea et al., 1973; Arocker-Mettinger et al., 1992; Mougeot et al., 1996; Chuck et al., 1997) and cerebellum 97 locations (Ntoukas et al., 2013).

As an animal model, the metacestode stage of *T. crassiceps* is usually maintained through serial intraperitoneal passages of cysts from the peritoneal cavity of infected to recipient naïve mice, where they reproduce asexually by budding (Freeman, 1962; Esch and Smyth, 1976; Good and Miller, 1976; Willms and Zurabian, 2010). Definitive hosts for *T. crassiceps* are foxes, groundhogs, wolves, and dogs, intermediate hosts include field rats, mice and other small rodents, (Loos-Frank, 2000).

Several strains of *T. crassiceps* have been reported, such as WFU (Everhart et al., 2004), HYG (Sally et al., 1976) or KBS (Dorais and Esch, 1969), isolated from natural infections. The cysticercus possesses an invaginated scolex, which evaginates when ingested by the definitive host and develops into the adult worm. In contrast, the ORF strain of *T. crassiceps*, isolated in 1962 (Freeman et al., 1962), lacks an invaginated scolex and has been considered a mutant strain. It has been proposed that ORF suffered a chromosomal loss that impairs scolex formation (Smith et al., 1972), thus producing sterile larvae that cannot develop into adult worms in the wild (Mount, 1968; Dorais and Esch, 1969); can only be propagated in the laboratory and are considered safe for human manipulation (Freeman, 1962).

In terms of genomic resources, the firsts genomes reported were for *Schistosoma japonicum* (Zhou et al., 2009), *S. mansoni* (Berriman et al., 2009). Since then, several genomes have been published including trematodes of medical importance such as, and *Clonorchis sinensis* (Wang et al., 2011; Huang et al., 2013). In 2013, the genomes of four cestodes: *Hymenolepis microstoma*, *Echinococcus granulosus*, *E. multilocularis* and *Taenia solium* were reported (Tsai et al., 2013) and a free living flatworm *Schmidtea mediterranea* (Grohme et al., 2018). Afterwards, the International Helminth Genomes Consortium started a project for genome characterization whose database (WormbaseParasite) already provides 45 Platyhelminthes and 19 cestodes (International Helminth Genomes Consortium, 2019).

In the last five years the genomes of other taeniidae have been reported: *T. asiatica*, *T. saginata* (Wang et al., 2016), *T. multiceps* (Li et al., 2018), *E. canadensis* (Maldonado et al., 2017), *E. oligarthrus* (Maldonado et al., 2019), *H. diminuta* (Nowak et al., 2019), *H. microstoma* (Olson et al., 2020). Availability of these genomes has increased the knowledge about gene numbers and composition of these organisms, improving our understanding of their biology and pathogenic mechanisms.

Here we report the high resolution characterization for the genome of *T*. *crassiceps* WFU strain and a comparison analysis against ORF strain. Both genomes were compared in order to find differences that could be related to the lack of scolex development. Our results provide evidence that the absence of scolex in the ORF strain was not the result of a chromosomal loss proposed elsewhere. Instead, it appears to be the result of subtle differences in the regulation of gene expression or unidentified mutations in specific genes.

## Materials and Methods

### Parasite Culture

Cysticerci from *T. crassiceps* strains WFU and ORF (**Supplementary Figure 1**) were propagated in the peritoneum of 4-week-old female BALB/c mice, which were inoculated intraperitoneally with 10 cysticerci and after 90 days were sacrificed for the recovery of the larvae. The cysticerci were thoroughly washed with sterile Phosphate Buffer Saline (PBS), pH 7.4, and then frozen at -70°C until use. The use and humanitarian handling of animals in this project was authorized by the Institutional Committee for the Care and Use of Laboratory Animals (CICUAL), UNAM with the register number 6329.

### Chromosome Spreading for *Taenia crassiceps* Karyotyping

The protocol for spreading chromosomes was adapted from (Guo et al., 2018 and Špakulová et al., 2011). Cysticerci recovered from the peritoneum of BALB/C mice with three months of infection were selected and placed in 16-well culture plates with RPMI-1640 media supplemented with 10% FBS and colchicine (0.25% w/v) during 5 h at 37°C. After incubation, cysticerci were washed twice with PBS and then placed in deionized water for 20 min and then fixed with a Methanol-Acetic acid solution (3:1) for 30 minutes. The chromosomes were spread by crushing the cysts between two slides with strong physical pressure. The slides were then frozen with liquid nitrogen and washed twice with PBS before being permeabilized with PBS-Triton X-100 (1%) for 5 min. Finally, the spread was stained with DAPI (1:500) for 20 minutes. Slides were mounted in Fluoroshield (Sigma) and observed under an inverted fluorescence microscope (Olympus IX71). Image analysis was made using FIJI software.

### DNA and RNA Isolation and Sequencing

Fresh cysticerci (both WFU and ORF strains) obtained from the peritoneum of mice were used for DNA and RNA extraction after three times washing in PBS, pH 7.4. A 1 mL of supernatant from the PBS recovered cysticerci was centrifuged at 12,000 g. The pellet was resuspended in 200 µL of Biofluid & Solid Tissue Buffer, added with 20 µL of Proteinase K and 2 µL de RNAsa (10 mg/mL), and incubated at 55°C for one hour. The lysis reaction was transferred using a bore-tip to a tissue grinder for 10 minutes and 1 mL of phenol was added and after 2 minutes, the solution was centrifuged at 12,000 g for another 2 minutes. A 350 µL volume of the supernatant was transferred to a clean tube and 1mL of chloroform was added. Chloroform addition and centrifugation was repeated twice and 250 µL of the supernatant were recovered and placed in 150 µL of RNAse-free water. DNA was extracted using the Zymo Quick-DNA™ HMW MagBead Kit (Catalog No. D6060) according to the vendor’s protocol. The same protocol was adapted for RNA extraction, skipping the 55°C incubation and harsher disruption using high-speed vortexing.

Different Illumina sequencing libraries were prepared using the extracted DNA from both WFU and ORF strains. Two Nextera XT DNA libraries, one with ~150 bp insert size and another with a ~500 bp insert size. Also, two Illumina Mate Paired libraries with 3-4 kb and 4-6 kb insert sizes, respectively. All libraries were prepared following the vendor’s protocols. For Oxford Nanopore sequencing, the DNA libraries for WFU and ORF DNA samples were prepared using the SQK-LSK109 using 1µg of DNA for each sample and following the vendor’s protocol. Sequencing was performed using a whole minION flow cell (v9.4) for each sample. Basecalling was done by the program Guppy v4.0.14 with default parameters and a high accuracy error model (dna_r9.4.1_450bps_hac.cfg).

RNA sequencing libraries were prepared using the Illumina TruSeq RNA Sample Prep Kit v2 (Illumina) following the manufacturer’s protocol. High quality RNA samples from WFU and ORF strains, three biological replicates for each one with RIN numbers ranging from 8 to 10 were used for NGS library construction. The libraries were sequenced using the Illumina NextSeq 500 platform with a paired-end configuration using 150 cycles (2x75 reads).

### Genome Assembly, Post-Assembly Improvement, Gene Prediction and Annotation

A *de novo* genome assembly for WFU strain was performed using a hybrid approach with two different sequencing technologies. The first assembly was obtained using the AllPaths-LG v.52448 (Butler et al., 2008) assembler using default parameters, with Illumina paired-end and mate pair libraries. Then, a second *de novo* assembly was obtained using Oxford Nanopore long reads and Canu v.1.9 (Koren et al., 2017) assembler. This assembly was corrected using two error correction tools, first Racon v1.3.1 (Vaser et al., 2002) with 3 iterations and later 3 iterations of Pilon v.1.23 (Walker et al., 2014), both used the illumina libraries employed in the first assembly. Finally, both assemblies were merged using the Ntjoin software v.1.0.8 (Coombe et al., 2020). The latest version of the genome can be found with the accession number JAKROA000000000, associated with the BioProject PRJNA807072.

For gene prediction, we first used RepeatModeler v1.0.11 (Smit et al., 2016) and RepeatMasker v4.08 (Smit et al., 2018) to softmasking the genome. We used Braker2 v2.1.6 (Brůna et al., 2021) which used 30,000 orthologous proteins that were obtained from *T. solium*, *E. multilocularis* and *E. granulosus* genomes and 10 Gb of RNAseq data from *T. crassiceps* to train AUGUSTUS v3.3.2 (Stanke et al., 2006) which run within the Braker pipeline. Briefly, we generated hints by mapping the RNAseq data using HISAT2 v2.2.1 (Kim et al., 2019) to generate a sorted BAM. Then, using the BAM file as hints, AUGUSTUS was trained along with the orthologous proteins to generate the gene predictions for *T. crassiceps*. The protein annotation was achieved using an in-house pipeline with the following steps: first we used Blastp v2.11.0 (Camacho et al., 2009) to align these against the curated protein database Swiss-Prot and assign the function of the protein with the greatest similarity. We used HmmScan v3.3.2 (Eddy, 2009) to search protein domains using the Pfam-A database, additionally we used SignalP v4.1 (Petersen et al., 2011) to identify annotated signal peptides. Finally, we enriched the annotation with the GO and KEGG database, assigning the GO terms associated with each protein and their relationship with metabolic pathways, respectively.

### SNPs Analysis

For variant calling and annotation, we used BWA v0.7.17 (Li and Durbin, 2009) to map the Illumina DNA sequencing of the ORF strain vs the WFU genome. Then we used Freebayes v1.2.0 (Garrison and Gabor, 2012) to analyze the BAM resulting from the previous alignment and generate the VCF variants file.

Annotation of the variants was performed using SnpEff v4.3t (Cingolani et al., 2012); the program takes the WFU annotation file in GFF format and its genome as a database and the resulting VCF from the variant call.

### Comparative Genomics

For the comparative genomic analysis, the genomes available at Genbank for *E. multilocularis* (GCA_000469725.3), *E. granulosus* (GCA_000524195.1), *E. canadiensis* (GCA_900004735.1), *T. solium* (GCA_001870725.1) and *H. microstoma* (GCA_000469805.3) were used. Also included was the *T. crassiceps* genome here described (JAKROA000000000). The Proteinortho v6.02 (Lechner et al., 2011) was used to obtain protein clusters of WFU genomes analyzed here with the following parameters: E-value for blast: 1e-05, minimum percent identity of best blast alignments: 25, minimum coverage of best blast alignments in percent: 50, minimum similarity for additional hits: 0.95. The results from the protein orthologous was also used to verify the annotation. Additionally, we used the Circos v0.69-8 (Krzywinski et al., 2009) package for circular data visualization.

### Differential Expression Analysis

We used Bowtie2 v2.3.4.3 (Langmead and Salzberg, 2012) to map the sequences from WFU and ORF RNAseq libraries to the coding sequences (CDS) of WFU strain. Subsequently, the counting matrices were obtained with Express v1.5.1 (Roberts et al., 2011) and finally the EdgeR v3.24.3 (Robinson et al., 2010) package was used to obtain the results of the differential expression analysis. We used a FDR cutoff value equal or less than 0.05 and Log-Fold Change value equal or greater than 1 to consider a gene as differentially expressed.

### Gene Ontology Analysis

We used the R package TopGO v2.46.0 (Alexa et al., 2006) to 256 perform enrichment analysis of the differentially expressed gene set and of those proteins that were unique to *T. crassiceps* in the comparison between orthologues.

## Results

### Genome Assembly and Curation

The complete genome for the *T. crassiceps* WFU strain was sequenced using genomic DNA obtained from cysts and reconstructed through a hybrid approach using Illumina and Nanopore technologies. The initial assembly produced 179 scaffolds for a total of 118.7 Mb. However, several of the shorter scaffolds presented repetitive patterns or high similarity to regions in larger scaffolds. After performing the comparison analysis at sequence level, we confirmed that 114 scaffolds belonged to telomeric or collapsed regions. Therefore, the curated final assembly we obtained included only 65 scaffolds for a total of 107.05 Mb. The statistics of the two genomic assemblies are shown in **Table 1**.

**Table 1:** *T. crassiceps* assemblies and genomic statistics.

	Initial assembly	Curated assembly
Total bases	118.70 Mb	107.05 Mb
Total number of scaffolds	179	65
N50/L50	6.70 Mb/5	10.55 Mb/4
N90/L90	186.59 kb/41	1.46 Mb/18
Ave. scaffold size	663.16 kb	1.65 Mb
Largest scaffold	19.05 Mb	19.05 Mb
BUSCO (eukaryota dataset) completeness	78.5%	78.5%
Predicted coding genes	12,089	10,585
Annotated proteins	9,603	9,603
rRNA and tRNA	348 rRNA (18S(22), 5.8S(305) y 28S(21)) y 43 tRNA	76 rRNA (18S(4), 5.8S(67) y 28S(5)) y 43 tRNA
Gene average length	5,533.7 bases	5,568 bases
Protein average length	523.17 residues	503.52 residues
Average exon number per gene model	7.76	8.78
Exon mean size	201.4	201.6 bases
Intron mean size	626.7	626.8 bases

### Noncontiguous Finished Resolution for *T. crassiceps* Genome and Comparative Genomics

**Figure 1:**
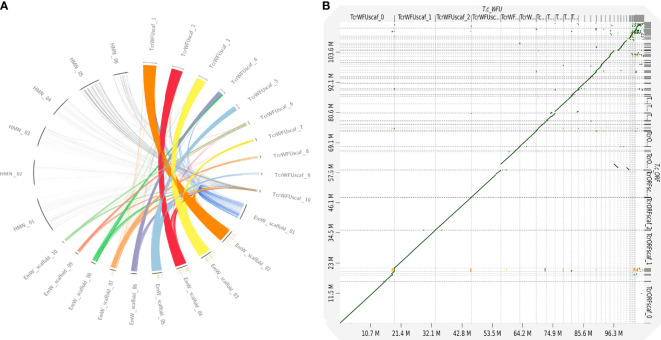
Comparative genomics between strains of *Taenia crassiceps* and with different cestode species. **(A)** Comparative genomics of three cestode genomes [*Hymenolepis microstoma* (HMN); *Echinococcus multilocularis* (EmW) and *Taenia crassiceps* WFU (TcrWFU)]. Scaffold sequences used for this comparison are available in the NCBI and WormBase databases. Genomic fragment names are those from the original source annotation **(B)** Dotplot comparison of WFU vs ORF strains at genomic level demonstrated a clear synteny discarding large structural variations.

We compared the ten largest scaffolds in the curated assembly of *T. crassiceps* with data from genomes of two closely related species with chromosome resolution assemblies. A CIRCOS plot comparison between *H. microstoma*, *E. multilocularis* and *T. crassiceps* is shown in **Figure 1A**. Three *T. crassiceps* scaffolds presented a similar degree of completeness and synteny to *E. multilocularis* chromosomes: scaffolds 1-3 of *T. crassiceps* vs. scaffolds 2, 4 and 3 from *E. multilocularis*, respectively. This suggested that these scaffolds might correspond to whole chromosomes. However, none of the compared *T. crassiceps* scaffolds showed a clear correspondence to *H. microstoma* scaffolds demonstrating a larger phylogenetic distance.

An extensive comparison of the identified proteins was carried out with five other species of cestodes to analyze their orthology with *T. crassiceps*. The proteomes of four taeniid species (*T. solium, T. crassiceps*, *E. multilocularis*, *E. granulosus* and *E. canadiensis*), as well as *H. microstoma* were compared (**Supplementary File 1**). A total of 4,665 orthologue groups were found in all species (cyclophyllidea core-genome); 5,221 were found in *T. solium, T. crassiceps*, *E. multilocularis*, *E. granulosus* and *E. canadiensis* (Taenidae core genome); 6,842 orthologue groups were shared between *T. solium* and *T. crassiceps* (*Taenia* core genome). Finally, 2,822 proteins were unique to *T. crassiceps* and did not cluster with other proteins in any other genome. Among these, 2,248 didn’t have a functional annotation.

### Genomic Variation Between *T. crassiceps* WFU and ORF Strains

Using the curated assembly of *T. crassiceps* WFU genome as a template, we evaluated the differences of the ORF strain. A dotplot analysis indicated that both assemblies were extremely close (**Figure 1B**); no differences among them were found that might suggest that a catastrophic genomic event happened in the ORF strain, such as the loss of a complete chromosome. As for the differences in both strains, using short read mapping with the Illumina sequencing, we found 2,487 site variants in ORF, distributed in 31 of the 65 scaffolds in the WFU curated assembly. Of these, 155 variants affected 147 protein coding genes resulting in missense or frameshift mutations. Variants were mainly single-base substitutions, insertions or deletions. Since long read sequencing was also done for the ORF strain, we searched for larger structural changes but none was found. All the information regarding the variants between the two strains can be found in the **Supplementary File 1**.

### WFU and ORF Karyotype Determination

It has been proposed that the lack of scolex in the ORF strain was due to an aneuploidy: a loss of a pair of chromosomes. Therefore, we also carried out a chromosome spreading to karyotype the metaphase nuclei of both strains. Interestingly the number 2n = 16 or 18 appeared with similar frequencies. This observation has been reported elsewhere (Špakulová et al., 2011). Whatever the number of chromosome pairs might be, we were unable to observe differences in both strains (**Supplementary Figure 2**). It is worth mentioning that the morphologic resolution we achieved did not allow us to observe if differences in the chromosome structure existed.

### Differential Expression Analysis Between ORF and WFU Strains of *T. crassiceps*

**Figure 2:**
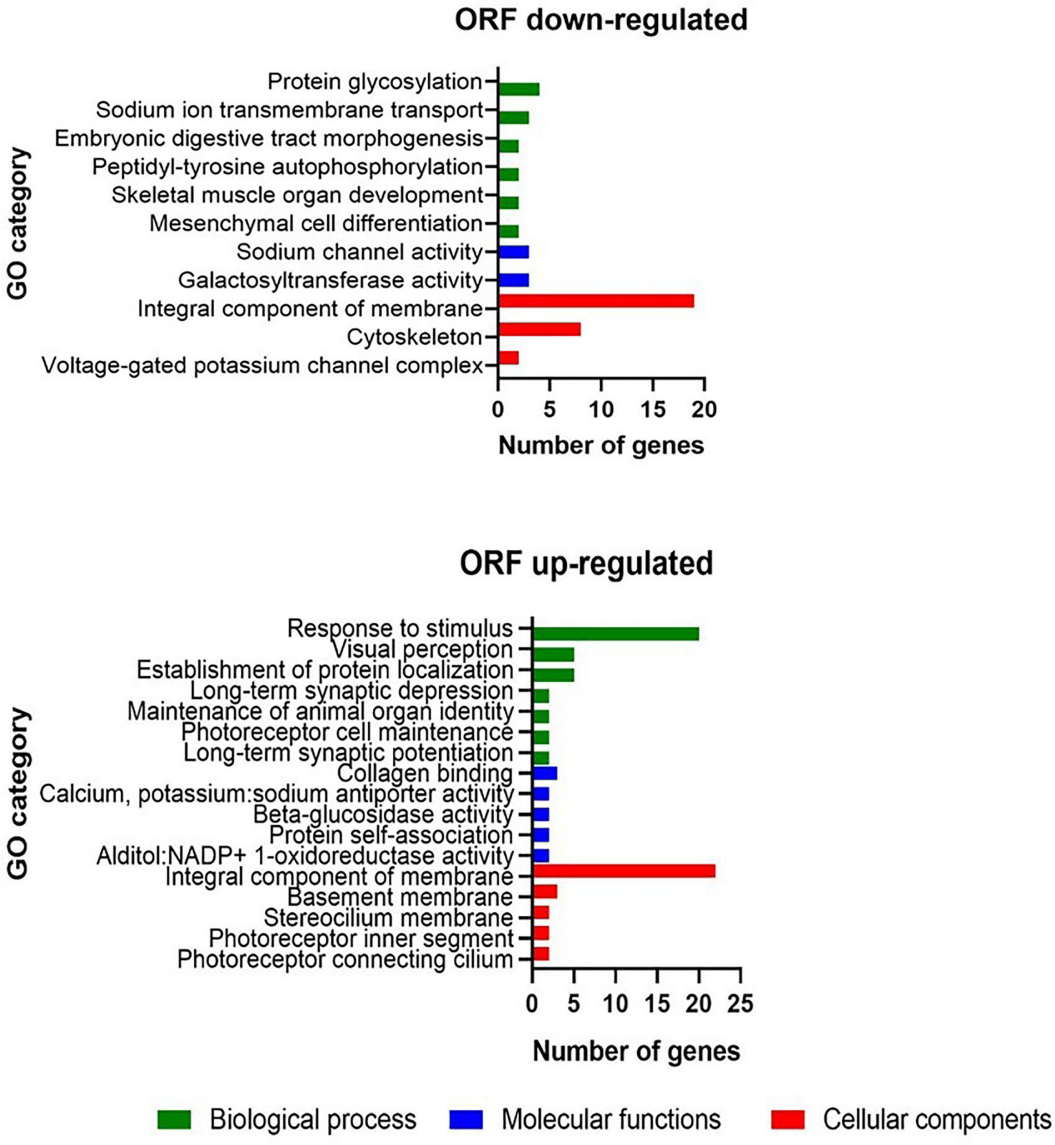
Gene ontology enrichment analysis in the *T. crassiceps* ORF strain with |logFc| >2 as threshold.

To elucidate the molecular basis of the phenotypic differences between WFU and ORF in relation with the lack of scolex development, we performed a differential expression analysis. A total of 1080 genes were found differentially expressed in the ORF strain. Among them, 627 and 453 genes were up and down-regulated, respectively (**Supplementary File 1**). Using a GO term enrichment analysis of down-regulated genes with |logFc| >2 as threshold (having functional annotation in ORF), different protein families were identified. Interestingly, some of them were related to embryonic digestive tract morphogenesis, mesenchymal cell differentiation, maintenance of animal organ identity, establishment of protein localization, among others (**Supplementary File 1**). Differentially expressed genes were also characterized by GO revealing a particular enrichment for both up and down-regulated genes related to integral membrane proteins in the cellular component category (**Figure 2**). In the same category, a considerable number of genes related to cytoskeleton were down-regulated. For up-regulated genes (**Figure 2**), in the biological processes category, most of the differentially expressed genes were grouped within response to stimulus. In total, 45 UF genes were also down regulated in ORF (**Supplementary File 1**).

### Differentially Expressed Genes and Genomic Variation

Analysis of genes with different expression levels whose coding sequence contained SNPs allowed the identification of 17 genes, including 5 of unknown function. Among the annotated genes, 3 were up-expressed and 7 were down-expressed in ORF, including some related with development, such as homeobox IRX-5 (TcrWFU_07074), rolling stone [rost] (TcrWFU_02139), as well as genes participating in the cell cycle: ubiquitin carboxyl terminal hydrolase 16 (TcrWFU_00103) and others involved in signal transduction pathways: Axin-1 (TcrWFU_07383) that participates in the Wnt/ß catenin pathway (**Supplementary File 1**).

## Discussion

The present work describes the characterized genomes and RNAseq results of two *T. crassiceps* strains (WFU and ORF), that are widely used as animal models for the study of human cysticercosis. The use of *T. crassiceps* as an adequate animal model seems justified in the light of our results of orthologue proteins in both species shown here; 6,842 orthologue groups were shared with *T. solium* demonstrating a high evolutionary closeness with *T. crassiceps*.

As mentioned above, the ORF strain lost the capacity to develop a scolex, structure that gives rise to the adult worm. It has been proposed that the absence of the scolex in the ORF strain was due to the loss of a pair of chromosomes (Smith et al., 1972). We undertook a genomic and transcriptomic characterization of these two strains as a way to approach this proposal. Initially, a dotplot analysis using the two assemblies showed minimal differences between both strains. Moreover, our karyotyping results did not reveal differences between the chromosome numbers in both strains, such as the absence of a whole chromosome pair (aneuploidy) reported previously (Smith et al., 1972). Even without a full chromosome resolution, the genomic information from both strains revealed no differences or massive structural changes that might support the loss of two chromosomes. Only minor differences were found in the genome drafts of both strains, including gene number, GC content and SNPs number. Instead, we hypothesize that the loss of scolex is the result of changes in the expression of genes involved in the development of this crucial structure in the ORF strain. In order to identify the network of genes resulting in the lack of scolex, we decided to analyze differences in gene expression (up or down-regulated) in the ORF strain. Considering that the scolex is the structure that gives rise to the adult stage of the parasite, we explored a diversity of genes related to embryonic development, formation of the anteroposterior axis, cell division, establishment of mitotic spindle orientation, among others.

The genome of *T. crassiceps* WFU strain was reconstructed using long and short read sequencing technologies. This allowed us to generate a noncontiguous high resolution genome, which was compared to the ORF strain and other cestode genomes with similar or higher resolution. According to the BUSCO completeness analysis, the reconstructed genome had a 78.5% completeness which is similar to the *E. multilocularis* genome (GCA_000469725.3). It is expected that genomes from parasitic species have a lower completeness due to a lack of proteins that are present in other free living eukaryotes. Interestingly, the ten largest fragments have a size similar to chromosomes in other species and five of them had a similar arrangement to chromosomes in *E. multilocularis*, which is a manually curated genome and a closely related species to *T. crassiceps*; both are included within the same family (Taenidae). Three *T. crassiceps* chromosomes (scaffolds 1-3) presented a very similar synteny to *E. multilocularis* but some of the comparisons suggested that other large fragments of *T. crassiceps* can also be arranged on the structure of other chromosomes in *E. multilocularis* (**Figure 1A**), although rearrangements cannot be ruled out. Therefore, other approaches like optical mapping can be used to resolve the assembly contiguity in the near future. Unfortunately, the current fragmentation of the *T. solium* genome, didn’t allow us to include it in the comparison. Our orthology analysis among cyclophyllideans, taeniids and *Taenia* genomes is of particular interest; it clearly shows increasing orthology within closer clades of tapeworms, supporting the basis of current taxonomy for this group of parasites. *T. solium* and *T. crassiceps* shared 6,842 orthologue groups, whereas 2,822 proteins were unique proteins for *T. crassiceps*; including 2,248 without functional annotation, not significant similarity with sequences in public databases. These unannotated proteins deserve further exploration to elucidate their role in this parasite. Finally, 574 unique proteins in *T. crassiceps* genome were included in a GO term enriched analysis (**Supplementary File 1**). From those unique proteins for *T. crassiceps*, we found 60 that were differentially expressed in ORF with respect to WFU. Interestingly, proteins without orthologues in other cestodes were related to molecular functions/biological processes such as arginyltransferase activity/protein arginylation; peptidase inhibitor activity/negative regulation of peptidase activity and glutathione peroxidase activity/response to oxidative stress. These ontologies suggest that some of the proteins may be involved in protease degradation/blocking or oxidative stress response. However, these and other results require further investigation and experimental assessment to confirm the role of those unique proteins in *T. crassiceps*.

From the total of 1080 genes, 843 had annotated function; our RNA-seq results indicated that 627 were upregulated and 453 genes were downregulated in ORF, in comparison to the WFU strain, considered as the wild-type strain in this work. Those changes in expression included zinc finger proteins, transcription factors, homeoboxes, components of the Wnt pathway, etc., that had been identified in previous studies on other cestode species (Olson et al., 2018).

As an example, in the ORF strain, down-regulated genes included homeobox B7 (HoxB7) (TcrWFU_04386), which is mainly related with processes of cell differentiation and segmentation in the adult stage; since they are presented in protoscoleces and in adult worms of *E. granulosus* (Dezaki et al., 2016). HoxB7 420 participates in processes such as cell proliferation and differentiation including 421 anterior/posterior pattern formation (Carè et al., 1999). Moreover, an important role of HoxB7 is related with morphological diversification of various body structures during development (Wellik, 2009). Regarding HoxB7, a differential expression has been demonstrated at different stages of development and structures in *E. granulosus* (*in vivo* and *in vitro*). Down-regulation of HoxB7 in ORF could be related to the absence of the scolex.

Another gene of interest related to development is frizzeld. Our results showed that frizzled (TcrWFU_07188) was down-regulated in the ORF strain. The BLAST result shows 100 and 89% identity with frizzeld-10 and frizzeld-4 of *E. granulosus*, respectively. Regarding the Wnt ligand, the frizzeld-4 gene (em-fz4) is expressed in dispersed cells in the germinal layer, becomes strongly up-regulated during brood capsule development, and is always restricted to the posterior-most region of the protoscolex throughout development in *H. microstoma* and *E. multilocularis* (Koziol et al., 2016). This finding in closely related organisms could explain why the gene is down-regulated, impairing formation of the scolex in *T. crassiceps* ORF strain.

On the other hand, down-regulated in the ORF strain (in comparison to WFU) whose function could be related to its inability to form a scolex, were seven genes involved in embryonic development and morphogenesis (**Supplementary File 1**). Among them, transcription factor 21 (TcrWFU_01181), a member of a subfamily of basic-helix-loop-helix proteins (bHLH) showed a high differential expression (LogFC = -2.02). This factor has been reported to play an important role in embryonic development of mesodermal tissues (Quaggin et al., 1998). Since the mesoderm gives rise to the notochord and the neural tube, it is also involved in the definition of the anteroposterior axis (Corallo et al., 2015). Another down-regulated protein (LogFC = - 2.23) in ORF is the Tyrosine protein-kinase src-1 (TcrWFU_10053), which together with Wnt, plays an essential role in embryonic development contributing to spindle orientation and endoderm specification in early development (Bei et al., 2002). Noteworthy, this gene is exclusively expressed in some head neurons of the nematode *Caenoharbditis elegans*, but not in its tail (Hirose et al., 2003). Finally, the other down-regulated gene in ORF is the fibroblast growth factor receptor-like 1 [(LogFC = -2.08) (TcrWFU_04317)], involved in the development of organisms and expressed in the anterior region (Lamb and Harland, 1995; Teven et al., 2014). This gene has been identified as up-regulated in larval and adult *H. microstoma* (HmN_000423900) (Olson et al., 2018).

Regarding proteins that are up-regulated in the ORF strain, one was the G protein-signaling modulator 2 [(LogFC = 2.43) (TcrWFU_07618)] belonging to a group of proteins that have the ability to activate G proteins. They are proteins involved in different biological processes, for example, cell division, establishment and organization of mitotic spindle also playing an important role in asymmetric cell divisions *via* NuMA (Du et al., 2001; Zhu et al., 2011; Kiyomitsu and Cheeseman, 2012). Some reports focus on G protein regulators (GPRs) in asymmetric positioning of the mitotic spindle in the early *C. elegans* embryo (Manning, 2003). Also up-regulated in ORF were Dystrobrevin-1 (TcrWFU_10303) and aquaporin-4 (TcrWFU_02255), which have also been found in the *Echinococcus* encystment process, playing a role of vasopressor and water reabsorption (Fan et al., 2020). These mechanisms have been related to the transport of proteins, carbohydrates, and other substances (Park and Kwon, 2015).

Proteins related to maintenance of animal organ identity were also upregulated in ORF (**Supplementary File 1**); among them, Fibronectins type III domain [(TcrWFU_10230, TcrWFU_10661, TcrWFU_10672 and TcrWFU_08319) (LogFC = 7.03, 8.13, 4.09 and 7.31)] are conserved proteins widely distributed in different species. Three of them have been reported as oncospheral proteins, although our results here shown demonstrated that these genes were also expressed in the larva. Also upregulated in ORF was an oncospheral fibronectin type III domain which has been used to develop a successful vaccine against porcine cysticercosis (Flisser et al., 2004; Gonzalez et al., 2005; Gauci et al., 2012). Finally, ORF cysticerci have a quicker reproductive rate in the peritoneal cavity of mice (Everhart et al., 2004; Fragoso et al., 2008; Willms and Zurabian, 2010), suggesting that upregulation of proteins related to cell proliferation and differentiation are involved. These findings allow focusing future studies to determine the causative agent of the lack of scolex in ORF.

### Genomic Variation Analysis of Differentially Expressed Genes

In addition to the genes that were found differentially expressed between the two strains, we identified a group of 12 other genes containing moderate and high impact SNPs, besides their differential expression state (**Supplementary File 1**). Three genes including high impact mutations that could affect their function, were found over-expressed in ORF. Protocadherin (TcrWFU_08480) which is necessary for cell-adhesion, might have an important role in the scolex formation. Other examples were the suppressor of tumorigenicity 7 protein (ST7) (TcrWFU_09775) and the adenylate cyclase type 9 (TcrWFU_09008), which are signaling proteins that have been well characterized in human development but are poorly studied in cestodes. If associated to scolex development, over expression of these regulators could be affected by mutations in those genes. For example, the gene that codes for Iroquois-class homeodomain protein IRX-5 (TcrWFU_07074), which is a transcription factor that has been associated with craniofacial development through modulating the migration of progenitor cells in the branchial arches of *Xenopus laevis* embryos; mutation of this gene prevents the correct craniofacial development causing dysmorphism (Bonnard et al., 2012). In ORF, we found that the SNP on IRX-5 contains a missense change (c.290T>C) with respect to WFU, suggesting that this mutation could affect its function and contribute to the lack in the development of the scolex.

Other gene with a high impact change (frameshift c.260delG) was the ubiquitin carboxyl-terminal hydrolase 16 [(UBP16) (TcrWFU_00103)], resulting in an amino acid deletion which might affect its important role in the deubiquitination of histone H2A, necessary for the progress of M phase in the cell cycle (Cai et al., 1999; Joo et al., 2007). On the other hand, UBP16 has been observed in *X. laevis* embryos, playing a role in the regulation of HOX genes through deubiquitination of histone H2A, which leads to the correct development of the anterior-posterior embryonic pattern (Joo et al., 2007).

Another downregulated gene in the ORF strain was Axin-1/DIX (TcrWFU_07383); which has been described as a protein with multiple functions, including the regulation of Wnt/β-catenin (cWnt) signaling pathway. Axin-1/DIX in ORF strain presented a SNP (c.1131delC) producing a frameshift of high-impact effect. Axins could interact with a β-catenin paralog and limit their accumulation; having highly segregated expression patterns along the anteroposterior axis in *E. multilocularis* and *H. microstoma*, this would indicate that β-catenin destruction complexes act during larval metamorphosis (Montagne et al., 2019). Dix domain is a family of proteins that form complexes with members of the disheveled protein family (Dvl) and Axin, acting as positive regulators of the Wnt signaling pathway (Shiomi et al., 2005). These complexes are involved in protein-protein interactions and are necessary for the ability of Dvl-1 and Axin to regulate the stability of β-catenin (Kishida et al., 1999).

Taken together, the above data suggest that the lack of scolex in the ORF strain of *T. crassiceps* could result from the action of multiple genes involved in the antero-posterior development of the parasite. Although a detailed scheme cannot be advanced at this point, something that can be ruled out is the proposal that the lack of scolex was due to the loss of complete chromosomes (Smith et al., 1972). Instead, our results suggest that it was due to a series of smaller changes, including single point mutations or differential expression of gene networks. Survival of this strain in natural conditions appears to be impractical. The role of 533 specific genes in the formation of the scolex could be approached through gene 534 silencing experiments on WFU cysts (Guerrero-Hernández et al., 2022).

Elucidation of the genomes of two strains of *T. crassiceps* will improve our knowledge about the genomic similarities and differences that exist between taeniid species, allowing novel approaches to a number of unsolved questions on tapeworm infections, including human neurocysticercosis caused by *T. solium*. We identified a core genome of conserved proteins for a group of cyclophyllidean cestodes, as well as some proteins that are unique to the *Taenia* genus and the *T. crassiceps* species. In this respect, improving the resolution of the *T. solium* genome is a must for future studies of comparative genomics within Taeniidae. Comparison between genomes might become a pipeline for the identification of potential targets and the design of new drugs, diagnostic tools or vaccines. Finally, availability of these high quality assemblies of *T. crassiceps* (WFU and ORF) can also contribute to defining the usefulness and limits of this animal model for the improvement of our understanding on human cysticercosis.

## Data Availability Statement

The datasets presented in this study can be found in online repositories. The names of the repository/repositories and accession number(s) can be found in the accession number JAKROA000000000, associated with the BioProject PRJNA807072.

## Ethics Statement

The animal study was reviewed and approved by Institutional Committee for the Care and Use of Laboratory Animals (CICUAL), Biomedical Research Institute-UNAM with the register number 6329.

## Author Contributions

KE performed the hybrid genome assembly, gene prediction, RNAseq differential expression and GO/KEGG enrichment analysis; PT performed initial sequencing, PT, DR-V and RJB maintained and isolated the cysts in mice, RJB, KE, DR-V, AC-G and JCC carried out the gene annotation, differential protein expression analysis, AS-F and KE did the comparative genomics (SNP and synteny analyses). JPL and AS-F conceived the project. RJB, KE and DR-V prepared the first draft and AS-F and JPL developed the final version. JPL provided the budget from grants. All authors contributed to the article and approved the submitted version.

## Funding

This report was supported in part by grants A1-5-11306 (CONACYT) and [IN 205820] PAPIIT-UNAM.

## Conflict of Interest

The authors declare that the research was conducted in the absence of any commercial or financial relationships that could be construed as a potential conflict of interest.

## Publisher’s Note

All claims expressed in this article are solely those of the authors and do not necessarily represent those of their affiliated organizations, or those of the publisher, the editors and the reviewers. Any product that may be evaluated in this article, or claim that may be made by its manufacturer, is not guaranteed or endorsed by the publisher.

## Supplementary Material

Supplementary Figure 1 Whole mount localization of proliferative cells in *T. crassiceps* ORF and WFU strains cysticerci. Cysts were labelled with EdU (red) or DAPI (blue) to reveal proliferative cells (phase S) and cell nuclei, respectively. **(A)** ORF cysts, **(B)** WFU cysts, and **(C)** WFU scolex. Scale bars in **(A, B)** represent 200 μm; bars in **(C)** represent 100 μm.

Supplementary Figure 2 Epifluorescence microscopy for karyotype determination in *T. crassiceps* WFU and ORF strains. Representative micrographs of the two different chromosome numbers observed in mitotic cytons from both strains of cysticerci: 2n=16, 2n=18.
