# Supplementary figures and images for: The Genomes of Two Strains of Taenia crassiceps the Animal Model for the Study of Human Cysticercosis
Source: Front Cell Infect Microbiol. 2022 May 10;12:876839. doi: 10.3389/fcimb.2022.876839 (PMC9128525; doi:10.3389/fcimb.2022.876839)

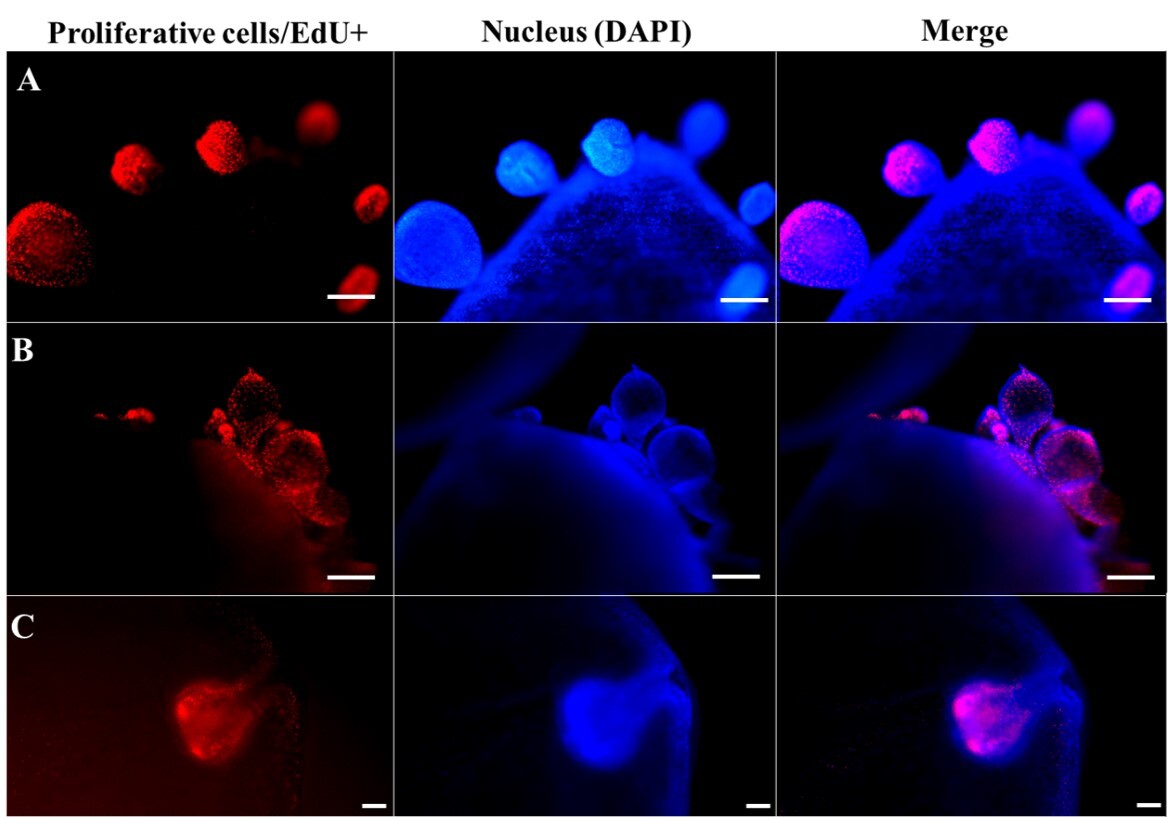

Supplement: Supplementary Figure 1 — Whole mount localization of proliferative cells in T. crassiceps ORF and WFU strains cysticerci. Cysts were labelled with EdU (red) or DAPI (blue) to reveal proliferative cells (phase S) and cell nuclei, respectively. (A) ORF cysts, (B) WFU cysts, and (C) WFU scolex. Scale bars in (A, B) represent 200 μm; bars in (C) represent 100 μm. [file Image_1.jpeg]

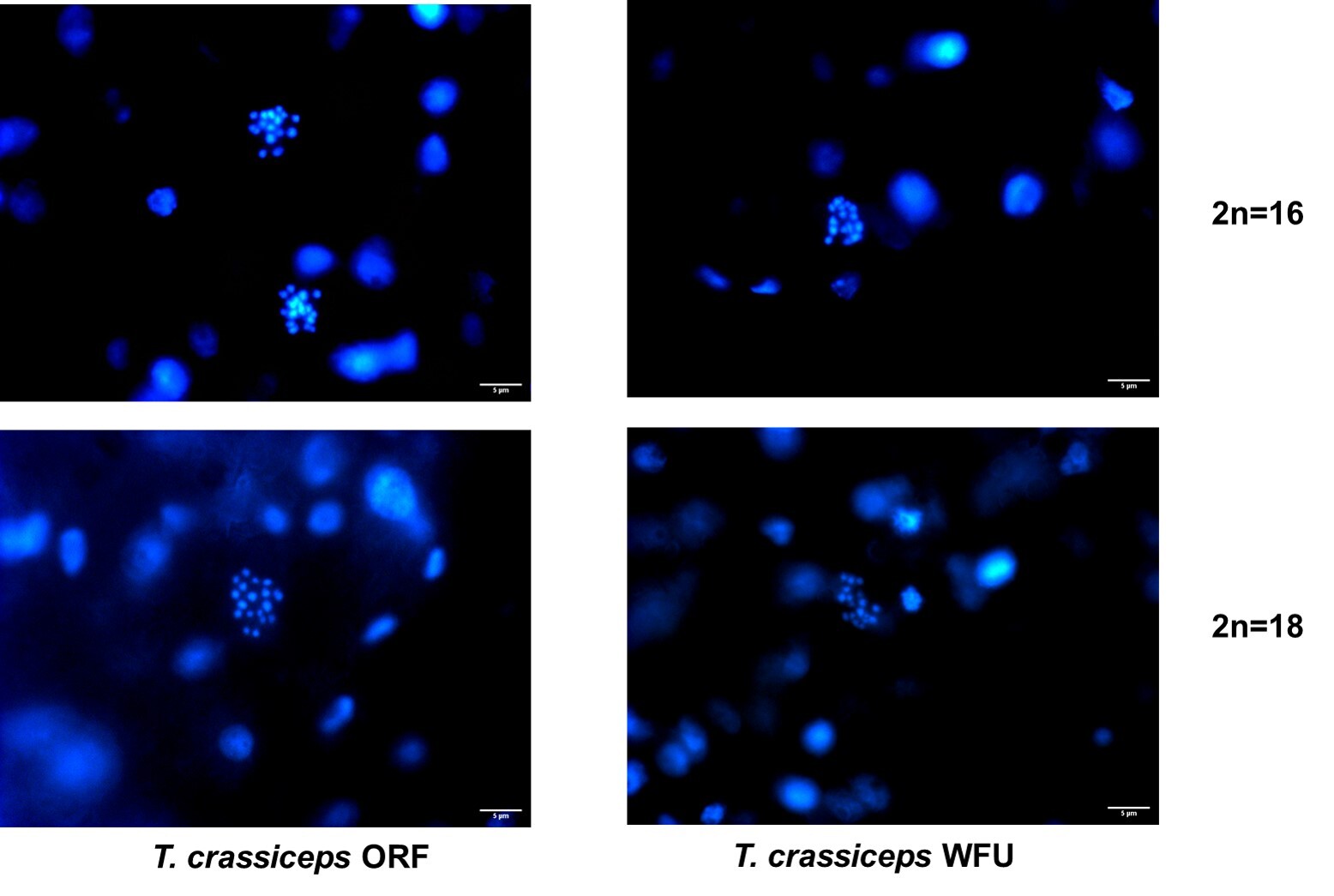

Supplement: Supplementary Figure 2 — Epifluorescence microscopy for karyotype determination in T. crassiceps WFU and ORF strains. Representative micrographs of the two different chromosome numbers observed in mitotic cytons from both strains of cysticerci: 2n=16, 2n=18. [file Image_2.jpeg]
